# Supplementary material for: Migration of Microplastics and Phthalates from Face Masks to Water
Source: Molecules. 2022 Oct 13;27(20):6859. doi: 10.3390/molecules27206859 (PMC9608222; doi:10.3390/molecules27206859)
Supplement: Supplementary file 1 [file molecules-27-06859-s001.zip › molecules-1917670-supplementary.pdf]

Supplementary material

MIGRATION OF MICROPLASTICS AND PHTHALATES FROM FACE MASKS TO WATER

Giuseppina Zuri, Bernat Oró-Nolla, Ana Torres, Angeliki Karanasiau, Silvia Lacorte*

[1] Institute of Environmental Assessment and Water Research of the Spanish Research Council (IDAEA-CSIC), Jordi Girona 18-26, 08034 Barcelona, Spain.

*Corresponding author: Silvia Lacorte; Ph.: +34 934006133; E-mail: slbqam@cid.csic.es

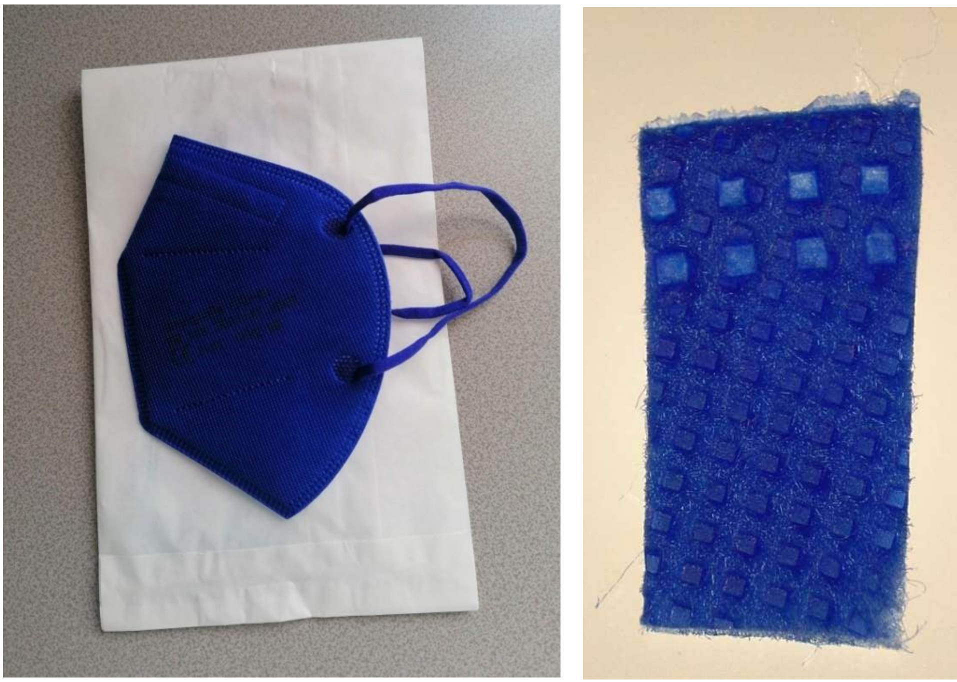

Figure S1 Face Mask FFP2 A a) intact and b) after disassembling it in layers.

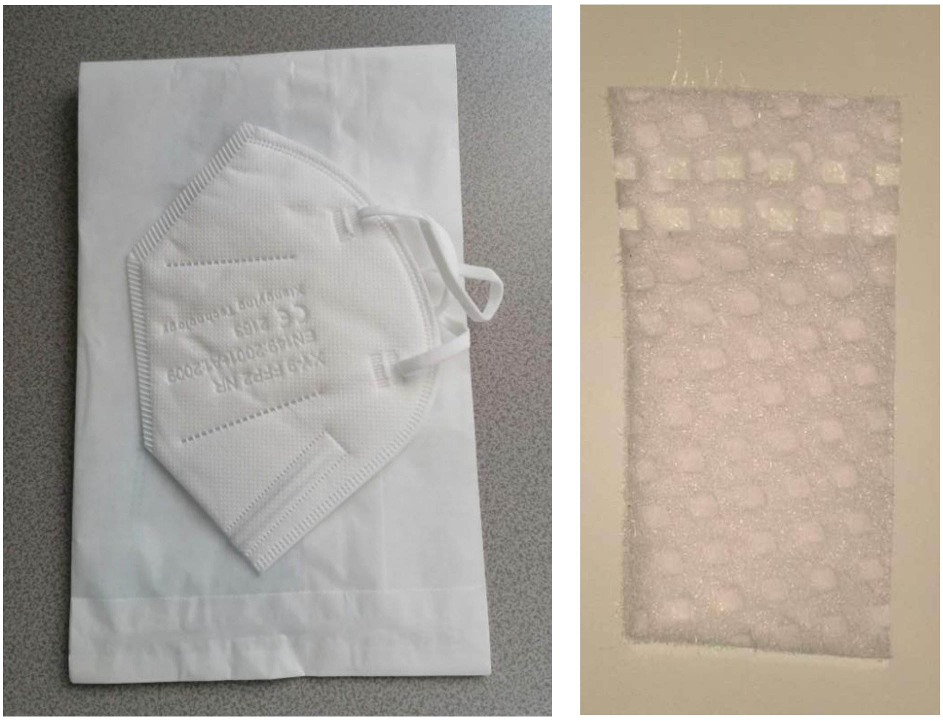

Figure S2 Face Mask FFP2 B a) intact and b) after disassembling it in layers.

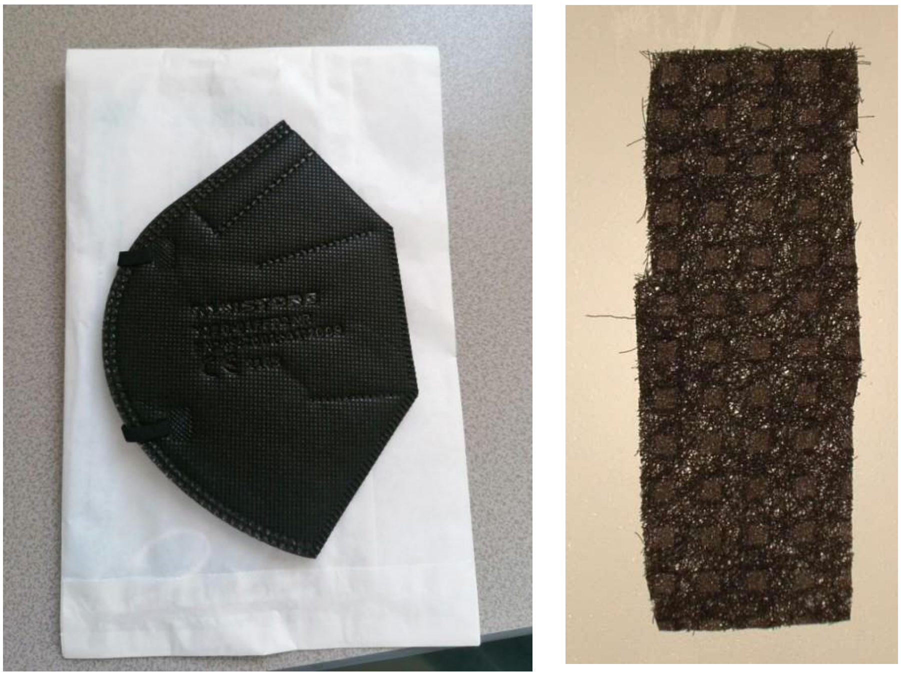

Figure S3 Face Mask FFP2 N a) intact and b) after disassembling it in layers.

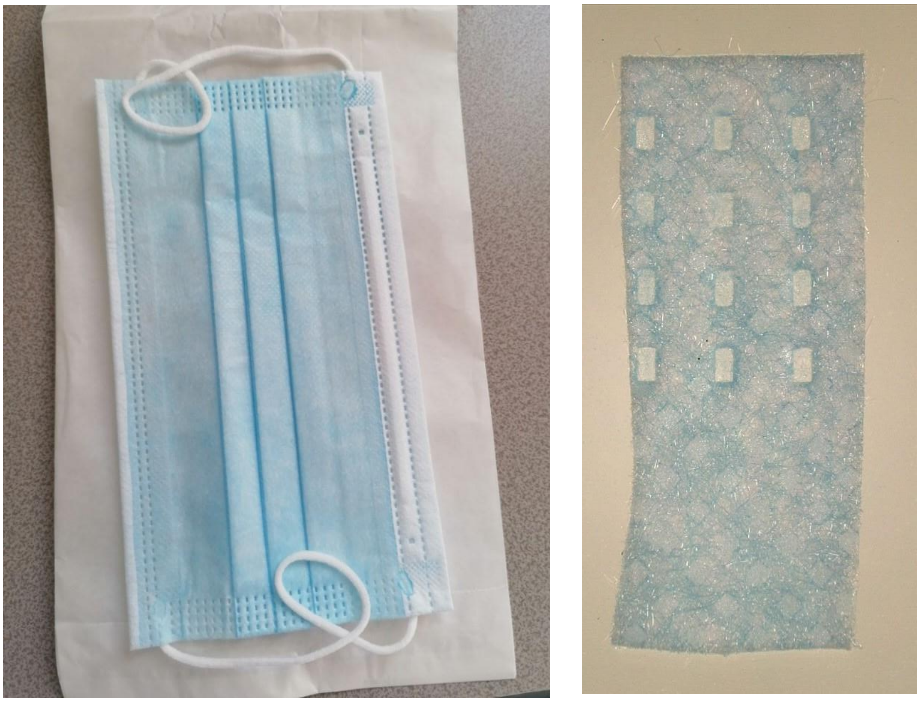

Figure S4 Surgical Mask Q a) intact and b) after disassembling it in layers.

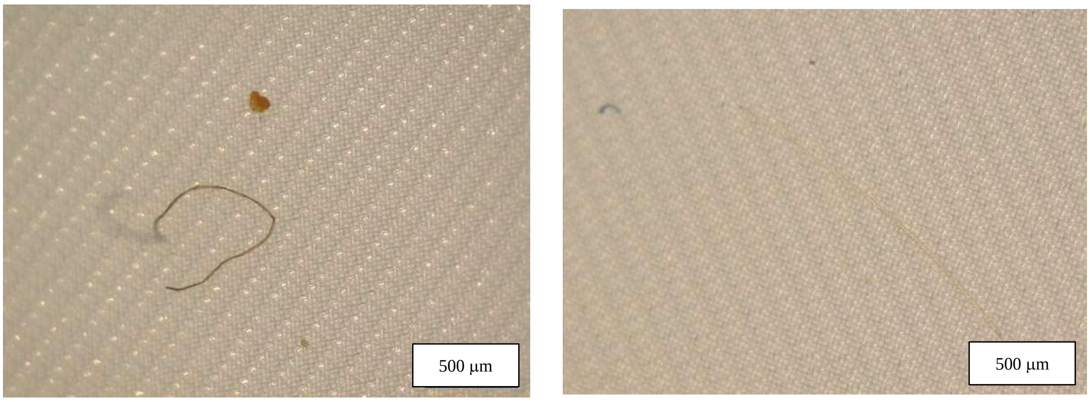

Figure S5 Microplastics detected in the filter of Blank 1.

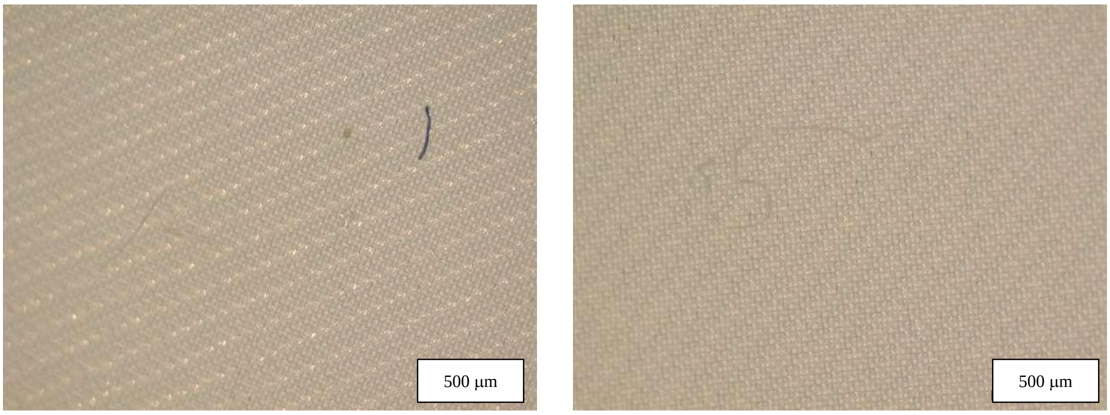

Figure S6 Microplastics detected in the filter of Blank 2.

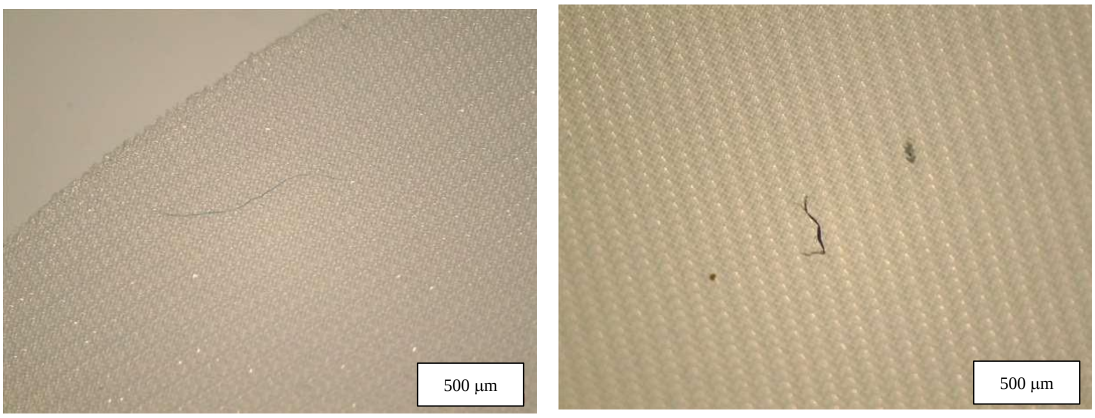

Figure S7 Microplastics detected in the filter of Blank 3.

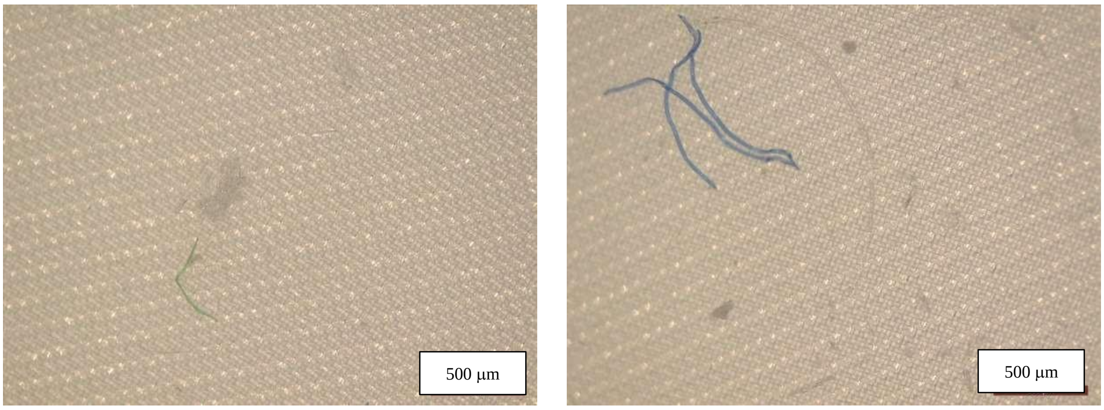

Figure S8 Microplastics detected in the filter of FFP2 A.

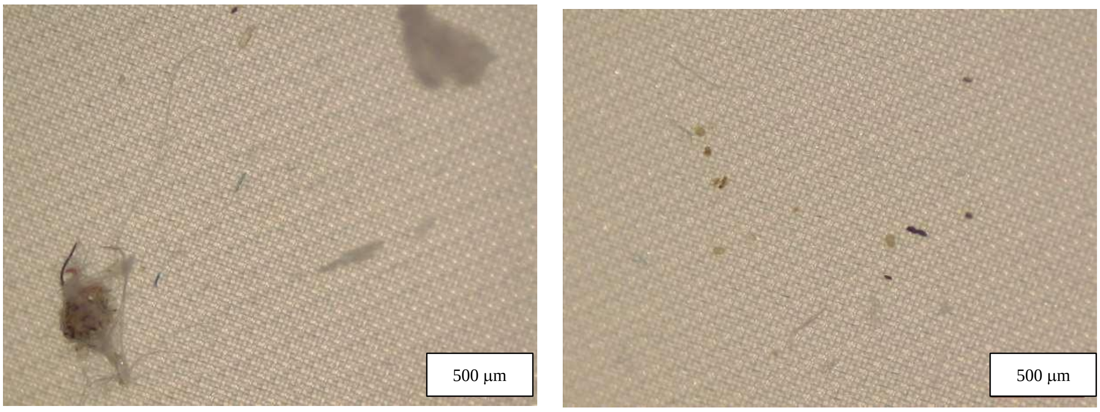

Figure S9 Microplastics detected in the filter of FFP2 B.

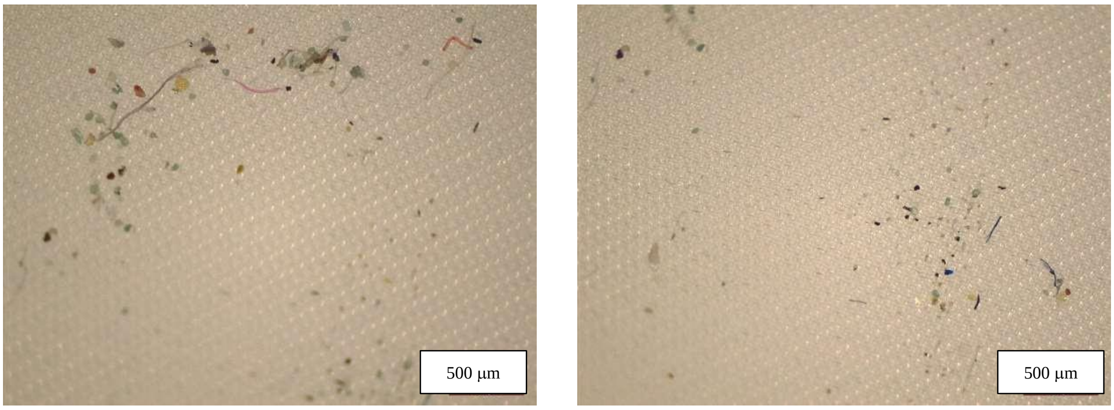

Figure S10 Microplastics detected in the filter of FFP2 N.

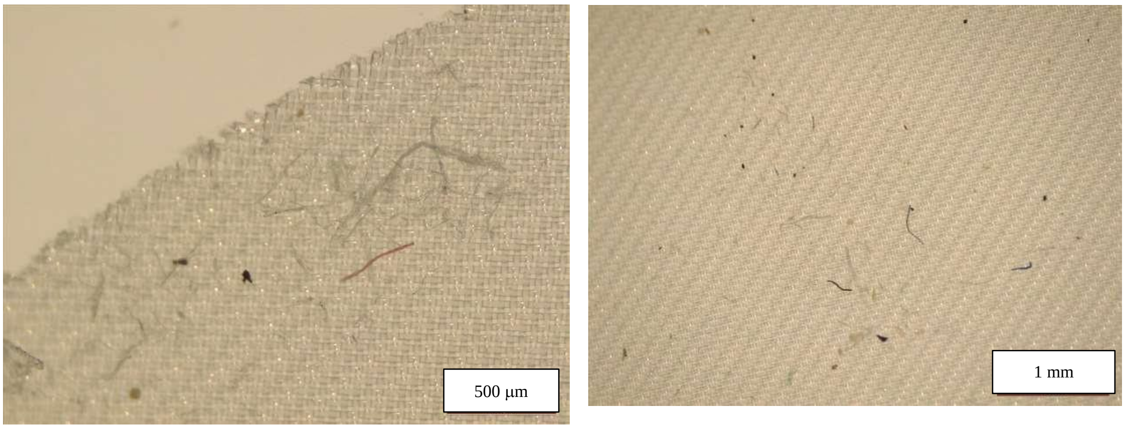

Figure S11 Microplastics detected in the filter of Surgical Mask.
